# Association of fetal growth trajectory with mitochondrial DNA copy number in the cord blood of newborns: evidence from a birth cohort

**DOI:** 10.3389/fped.2025.1569702

**Published:** 2025-06-09

**Authors:** Kai Chen, Junwei Li, Luli Xu, Xiaoxuan Fan, Zhongqiang Cao, Lulu Song, Youjie Wang, Chao Xiong, Aifen Zhou

**Affiliations:** ^1^ Data Center, Wuhan Children's Hospital (Wuhan Maternal and Child Health Care Hospital), Tongji Medical College, Huazhong University of Science and Technology, Wuhan, China; ^2^ Institute of Maternal and Child Health, Wuhan Children's Hospital (Wuhan Maternal and Child Health Care Hospital), Tongji Medical College, Huazhong University of Science and Technology, Wuhan, China; ^3^ Department of Obstetrics, Wuhan Children's Hospital (Wuhan Maternal and Child Health Care Hospital), Tongji Medical College, Huazhong University of Science and Technology, Wuhan, China; ^4^ Department of Maternal and Child Health, School of Public Health, Tongji Medical College, Huazhong University of Science and Technology, Wuhan, China

**Keywords:** gestational ultrasound measurements, fetal growth trajectory, intrauterine growth pattern, mitochondrial DNA, mtDNA, mitochondrial DNA copy number, mtDNAcn

## Abstract

**Objective:**

Mitochondrial DNA copy number (mtDNAcn), an indicator of mitochondrial damage and dysfunction, is widely used in research related to growth and metabolic health. While fetal intrauterine growth has been reported to impact further metabolic health, there is limited evidence regarding the relationship between fetal growth patterns and newborn mtDNAcn, especially in infants with normal birth weights, where varying fetal growth patterns can occur despite having the same birth weight. Therefore, this study aimed to examine the association between fetal growth trajectory and neonatal mtDNAcn among normal birth weight infants.

**Methods:**

A total of 556 mother–infant pairs from a birth cohort in Wuhan, China, were included in the study. Ultrasound measurements (biparietal diameter, head circumference, abdominal circumference, and femoral length) were taken at 16, 24, 30, and 37 weeks of pregnancy and converted to Z-scores per WHO standards, and the fetal growth trajectory was fitted by the group-based multi-trajectory model. Cord blood was collected at birth, and mtDNAcn in cord blood was quantified via real-time fluorescent quantitative PCR. A generalized linear model was used to explore the associations of fetal growth pattern or birth weight with neonatal mtDNAcn.

**Results:**

Three distinct patterns of fetal growth trajectory were identified, namely, “consistently low” (*n* = 144, 25.9%), “moderate” (*n* = 304, 54.7%), and “high-falling” (*n* = 108, 19.4%). Compared with the “moderate” intrauterine growth pattern, the “consistently low” intrauterine growth pattern was associated with lower neonatal mtDNAcn among male newborns, with a reduction of 22.55% (95% CI: −39.19%, −1.37%; *p* = 0.039). No significant association was detected between the intrauterine growth pattern and mtDNAcn among girls.

**Conclusions:**

Our findings indicate that different intrauterine growth patterns are present in fetuses with normal birth weights. In male infants, the “consistently low” intrauterine trajectory pattern was associated with decreased neonatal mtDNAcn. The effective detection of and intervention in fetal intrauterine growth patterns may help prevent metabolic health events early in life.

## 1 Introduction

Birth weight is widely recognized as a key indicator of fetal intrauterine growth and has been established as an important marker for assessing postnatal health risks (1). Importantly, however, infants with the same birth weight may have different fetal growth trajectories (2, 3). Relying solely on a single measurement at birth may not fully capture the complexity of fetal growth trajectories and their implications for research findings (2). Epidemiological studies have demonstrated that inadequate fetal growth and development not only impact health at birth but also increase susceptibility to multiple metabolic health-related diseases later in life (4, 5). Serial ultrasound examinations conducted throughout pregnancy are vital for ensuring accurate gestational age determination while evaluating fetal growth (6). These examinations are gradually being used to study dynamic fetal growth patterns alongside their associated health outcomes.

Mitochondria are crucial organelles in eukaryotic cells, play pivotal roles in energy supply and cell proliferation, and are closely associated with intrauterine and postnatal growth and metabolic health (7–9). Furthermore, mitochondria are a significant source of intracellular reactive oxygen species (ROS) and are particularly vulnerable to oxidative stress (10, 11). Owing to the absence of protective histones and an efficient DNA repair system, mitochondrial DNA (mtDNA) is highly susceptible to oxidative damage and has a high mutation rate (10). Mitochondria counteract these mutations by increasing the rate and quantity of mitochondrial gene duplication. However, when the extent of damage exceeds the compensatory capacity, it ultimately leads to a decline in mtDNA content (9, 10). Consequently, alterations in mtDNA content can serve as indicators of mitochondrial impairment and dysfunction. Recently, DNA copy number (mtDNAcn), an indicator of mitochondrial DNA content, has been extensively utilized in clinical and epidemiological studies (11, 12). While a few studies (13–19) have reported an association between birth weight and cord blood or placental mtDNAcn, these studies had small sample sizes and inconsistent findings. Furthermore, these studies focused primarily on infants with low birth weights or intrauterine growth restrictions rather than those with normal birth weights, who constitute the majority of new-borns.

On the basis of a birth cohort, this study aimed to identify the intrauterine fetal growth trajectory pattern on the basis of routine ultrasound parameters and to explore the associations between different patterns and mtDNAcn in the cord blood of newborns.

## 2 Methods

### 2.1 Study design and population

All participants were recruited at Wuhan Children's Hospital (Wuhan Maternal and Child Health Hospital). Between July 2014 and October 2015, a total of 565 participants met the inclusion criteria for this study: (1) singleton pregnancy and recruitment at less than 16 weeks of gestation; (2) prenatal care, including at least three ultrasound examinations at the study hospital; and (3) term delivery (37–41 weeks) at the study hospital with umbilical cord blood donation. Participants were excluded if they had a diagnosis of low birth weight (<2,500 g), intrauterine growth retardation (IUGR, defined as birth weight < 3rd percentile) (*n* = 6), or poor-quality cord blood testing (*n* = 3). Finally, 556 participants were included in the analysis. Of these, 290 (52.2%) completed all four scheduled ultrasound examinations (16, 24, 30, and 37 weeks). Missing data patterns were as follows: 92 (16.5%) missed the 16-week measurement, 69 (12.4%) missed the 24-week measurement, 97 (17.4%) missed the 30-week measurement, and 8 (1.4%) missed the 37-week measurement.

Informed consent was obtained from each participant at the time of recruitment into the study. The research protocol was approved by the ethics committees of Wuhan Children's Hospital (Wuhan Maternal and Child Healthcare Hospital) and Tongji Medical College, Huazhong University of Science and Technology (approval number: 2010009).

### 2.2 Fetal ultrasound measurements during pregnancy

Each participant typically underwent ultrasound examinations conducted by a professional ultrasound doctor at approximately 16 (range, 13–18) weeks, 24 (range, 21–25) weeks, 30 (range, 28–32) weeks, and 37 (range, 36–40) weeks of gestation. The fetal ultrasound parameters measured included the biparietal diameter (BPD), head circumference (HC), abdominal circumference (AC), and femur length (FL). The gestational age was calculated on the basis of the last menstrual period (LMP). If the gestational age determined by ultrasound differed from that based on the LMP by more than 7 days, the ultrasound-corrected gestational age was used.

### 2.3 Assessment of mtDNA content

Umbilical cord blood was collected immediately after delivery via ethylenediaminetetraacetic acid (EDTA) tubes. The samples were centrifuged to separate the plasma and blood cells, which were then frozen at −80°C for further analysis. DNA extraction from umbilical cord blood leukocytes was performed via a Wizard® Genomic DNA Purification Kit (Promega Corporation, Madison, WI, USA). The concentration and purity of the DNA samples were assessed via a NanoDrop™ 1,000 Spectrophotometer (Thermo Fisher Scientific, USA), with a standard A260/A280 ratio of 1.8–2.0.

The relative content of mtDNA in umbilical cord blood was determined via real-time quantitative polymerase chain reaction (qPCR) as described in a published study with some modifications (20). The relative content of mtDNA was calculated by dividing the mitochondrial gene copy number (ND1) by the single-copy nuclear control gene [human β-globulin (hbg)]. qPCR was performed on a 384-well plate using the ViiA™ 7 Dx Real-Time PCR System (Applied Biosystems, USA). The sequences of the primers used were as follows: forward primer ND1-F, 5′-CCCTAAAACCCGCCACATCT-3′; reverse primer ND1-R, 5′-GAGCGATGGTGAGAGCTAAGGT-3′; forward primer hbg-f, 5′-GTGCACCTGACTCCTGAGGAGA-3′; reverse primer hbg-r, 5′-CCTTGATACCAACCTGCCCAG-3′. Each reaction mixture, totaling 10 μl, was composed of 5.0 μl of KAPA SYBR® FAST qPCR Kit Master MIX 2×, 1.0 μl of 10 ng/μl genomic DNA, 0.2 μl of 10 μM ND1-F (hbg-f), 0.2 μl of 10 μM ND1-R (hbg-r), and 3.6 μl of RNase-free water. Each sample was tested in triplicate, and standard deviations for the cycle threshold (Ct) value were maintained at or below 0.30. The measurements were repeated if the coefficient of variation (CV) of the Ct value exceeded 0.30.

The amplification conditions were as follows: a prerun step of 50°C for 2 min and 95°C for 3 min to activate DNA polymerase, followed by 40 cycles of denaturation at 95°C for 3 s and annealing/extension at 60°C for 30 s. Melt curve analysis was conducted at the end of each run to confirm amplification specificity. To generate the standard curve, reference DNA derived from a pooled sample of 50 randomly selected samples was used, with five-point DNA concentrations ranging from 0.4 ng/μl to 104 ng/μl achieving a coefficient of determination (*R*^2^) > 0.99. All the samples were tested in triplicate, and the coefficients of variation (CVs) for interplate and intraplate values calculated from the Ct values of the single-copy genes were 3.8% and 2.8%, respectively.

### 2.4 Covariates

During recruitment, participants completed a structured questionnaire, including demographic information, lifestyle information, obstetric history, and health information. Information regarding gestational diabetes mellitus (GDM), pregnancy-induced hypertension (PIH), and newborn birth was extracted from hospital electronic medical records. Pre-pregnancy BMI (kg/m^2^) was calculated as pre-pregnancy weight (kg) divided by the square of pre-pregnancy height (m^2^). Maternal passive smoking during pregnancy was self-reported and was defined as exposure to passive smoking during the six months before pregnancy. The covariates analyzed in this study included maternal age (years), pre-pregnancy BMI (kg/m^2^), education level (college or above/high school/junior high school or below), parity (nulliparous/multiparous), GDM (yes/no), PIH (yes/no), passive smoking during pregnancy (yes/no), newborn sex and gestational age at birth.

### 2.5 Statistical analysis

Group-based multi-trajectory modelling (GBMTM), a specialized form of finite mixture modeling, is designed to identify subgroups of individuals with similar trajectories of the measure of interest in multivariate longitudinal data (21, 22), which has been increasingly used in clinical studies in recent years (22), including identifying fetal growth trajectories (23, 24). In this study, the GBMTM was applied to identify subgroups of participants with similar trajectories of fetal ultrasound measurements, which can capture the correlations and potential synergistic effects of BPD, HC, AC, and FL. According to World Health Organization (WHO) standards (25), all fetal ultrasound parameters are converted to Z-scores on the basis of gestational age at the time of measurement and then fitted to the multi-trajectory model. The optimal number of trajectory subgroups and trajectory morphology were satisfied according to the following criteria (21, 22, 26): (1) smaller value in the Akaike information criterion and Bayesian information criterion; (2) each trajectory group comprising more than 5% of the total sample size; (3) average posterior probability for each trajectory group exceeding 0.7; and (4) interpretability of the trajectory.

Continuous variables with a normal distribution are expressed as the means ± standard deviations (SDs) and were compared via t tests. Continuous variables with a skewed distribution are expressed as medians (interquartile ranges, IQRs) and were compared via the Wilcoxon rank sum test. Categorical variables are expressed as frequencies (percentages) and were compared via chi-square tests or Fisher's exact tests. Analysis of variance (ANOVA) was used to compare the differences in ultrasonic parameters or birth weight among the three trajectory patterns. Pairwise comparisons of birth weight among the three fetal growth trajectory groups were performed using *post-hoc* tests with Bonferroni correction. The generalized linear model was used to assess the association of fetal growth trajectory patterns with neonatal mtDNAcn. The fetal trajectory pattern of moderate growth accounted for the largest proportion and was used as the reference group. Owing to its skewed distribution, Lg-transformed mtDNAcn was included in the regression model as an outcome variable. The beta coefficient (β) and 95% confidence intervals (CIs) were estimated for mtDNAcn according to the trajectory patterns. For easier interpretation of the model, we used (10^β^—1) × 100% to estimate the percentage change in mtDNAcn for different fetal trajectory patterns compared with the reference group and (10^β±1.96 ×SE^—1) × 100% to estimate the 95% CI. In addition, newborn birth weight was analyzed as an outcome variable to assess the association of birth weight (per 1,000 g increase) with neonatal mtDNAcn. The multivariate logistic regression model was employed to assess the association between fetal growth trajectory patterns and mtDNAcn tertile categories (first, second, and third tertile), and to compute odds ratios (OR) along with their 95% CI. Covariates such as maternal age, pre-pregnancy BMI, maternal education level, parity, GDM, PIH, passive smoking during pregnancy, newborn sex, and gestational age at birth were selected according to previous studies (27–29) and adjusted for in the analysis. In addition, we performed stratified analysis to evaluate the associations of fetal growth trajectory with and newborn mtDNAcn across different sexes, and found no statistically significant interaction between sex and trajectory patterns in all the models.

All the statistical analyses were conducted via SAS 9.4 (SAS Institute Inc., Cary, NC), with GBMTM performed via PROC TRAJ macros. *P* values < 0.05 were considered statistically significant.

## 3 Results

The study included 556 mother–newborn pairs (Table 1). The mean maternal age at delivery was 28.6 ± 3.2 years, and the pre-pregnancy BMI was 20.9 ± 2.8 kg/m^2^. Most of the mothers had a college degree or above (78.4%), were nulliparous (86.5%), and reported no passive smoking during pregnancy (67.1%). GDM was present in 7.9% of mothers, whereas PIH affected 2.2%. For the newborns, the gestational age at birth was 39.40 ± 1.01 weeks, the birth weight was 334 ± 0.39 kg, and the relative mtDNAcn in cord blood was 1.21 (0.69–2.16). There was a significant difference in gestational age (*p* = 0.012) and birth weight (*p* = 0.047) between male and female infants but not in mtDNAcn (*p* = 0.657).

**Table 1:** Descriptive characteristics of the participants.

Characteristics	Total (*n* = 556)	Male newborns (*n* = 292)	Female newborns (*n* = 264)	*p* value
Mothers				
Maternal age (years)	28.59 ± 3.23	28.79 ± 3.41	28.36 ± 3.01	0.110
Pre-pregnancy BMI (kg/m^2^)	20.92 ± 2.83	21.00 ± 2.87	20.83 ± 2.80	0.517
Maternal education level				0.276
College or above	436 (78.4)	233 (79.8)	203 (76.9)	
High school	90 (16.2)	41 (14.0)	49 (18.6)	
Junior high school or below	30 (5.4)	18 (6.2)	12 (4.5)	
Parity				0.058
Nulliparous	481 (86.5)	245 (83.9)	236 (89.4)	
Multiparous	75 (13.5)	47 (16.1)	28 (10.6)	
GDM				0.973
No	512 (92.1)	269 (92.1)	243 (92.0)	
Yes	44 (7.9)	23 (7.9)	21 (8.0)	
PIH				0.321
No	544 (97.8)	284 (97.3)	260 (98.5)	
Yes	12 (2.2)	8 (2.7)	4 (1.5)	
Passive smoking during pregnancy				0.782
No	373 (67.1)	191 (65.4)	182 (68.9)	
Yes	183 (32.9)	101 (34.6)	82 (31.1)	
Newborn				
Gestational age (weeks)	39.40 ± 1.01	39.30 ± 0.98	39.52 ± 1.03	0.012
Birth weight (kg)	3.34 ± 0.39	3.38 ± 0.40	3.31 ± 0.37	0.047
Relative mtDNAcn in cord blood^a^	1.20 (0.69–2.15)	1.19 (0.78–2.04)	1.21 (0.62–2.35)	0.657

^a^ The relative mtDNAcn in cord blood was computed by the ratio of mitochondrial DNA copy numbers to the single-copy nuclear control gene [human β-globulin].

mtDNAcn, mitochondrial DNA copy number; BMI, body mass index; PIH, pregnancy induced hypertension; GDM, gestational diabetes mellitus.

**Figure 1:**
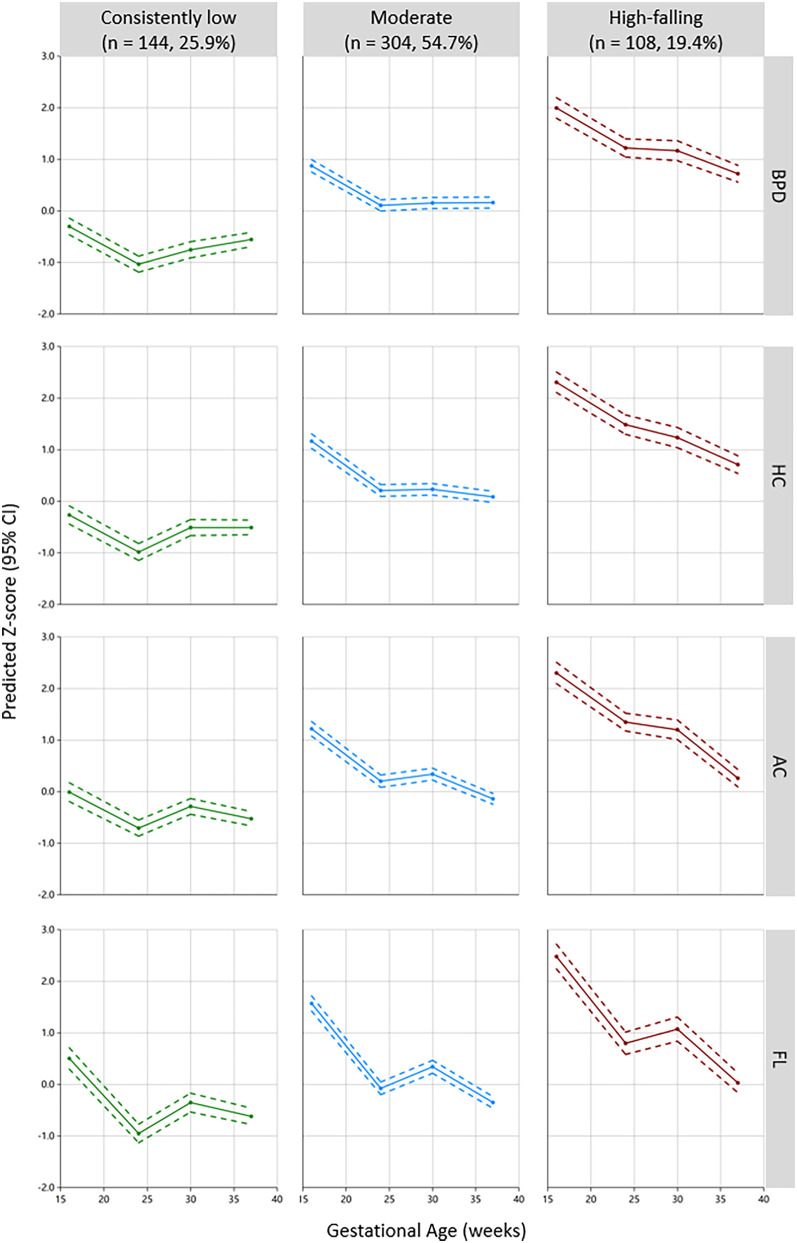
Three-group multi-trajectory model of fetal growth on the basis of the ultrasound parameters BPD, HC, AC, and FL. BPD, biparietal diameter; HC, head circumference; AC, abdominal circumference; FL, femur length.

Among the 556 subjects, the GBMTM identified three distinct fetal growth trajectories integrating BPD, HC, AC, and FL (Figure 1). Fetuses in the “consistently low” (*n* = 144, 25.9%) group showed consistently low intrauterine growth, with a trajectory predominantly below the Z-score of zero. The “moderate” group (*n* = 304, 54.7%) displayed an above-average growth level, serving as the reference group in subsequent analyses. The “high-falling” fetal growth trajectory (*n* = 108, 19.4%) was characterized by a high growth level (Z-scores > 2.0) at 16 weeks of gestation, gradually declining thereafter, still surpassing the “moderate” group at 37 weeks of gestation near delivery. The actual measured values, z-scores of ultrasound examination four times during pregnancy and birth weight by different fetal growth trajectory patterns are displayed in Table 2. There were statistically significant differences in each ultrasound measurement index across the different groups at the four pregnancy times. The average birth weights of the “consistently low”, “moderate”, and “high fitting” fetal growth trajectory groups were 3200.07 ± 353.41 g, 3339.24 ± 386.80 g, and 3552.04 ± 345.17 g, respectively, and there was a statistically significant difference in birth weight between any two groups (Table 2).

**Table 2:** Fetal ultrasound parameters during pregnancy and birth weight by different fetal growth trajectory patterns.

Ultrasound parameters or birth weight	Fetal growth trajectory patterns		
	Consistently low (*n* = 144)	Moderate (*n* = 304)	High-falling (*n* = 108)
Ultrasound parameters at 16 weeks of gestation			
BPD, mm	32.83 ± 2.96	34.42 ± 3.65	36.84 ± 3.48
HC, mm	123.65 ± 9.71	130.59 ± 10.25	136.44 ± 11.26
AC, mm	105.13 ± 8.75	111.69 ± 9.34	117.88 ± 10.06
FL, mm	20.40 ± 2.23	22.03 ± 2.64	23.37 ± 2.59
BPD for Z-score	−0.28 ± 0.83	0.89 ± 0.79	2.04 ± 0.95
HC for Z-score	−0.30 ± 0.97	1.17 ± 1.00	2.34 ± 0.98
AC for Z-score	−0.06 ± 1.00	1.21 ± 0.96	2.33 ± 0.99
FL for Z-score	0.43 ± 1.12	1.63 ± 1.17	2.54 ± 1.17
Ultrasound parameters at 24 weeks of gestation			
BPD, mm	57.10 ± 2.11	59.61 ± 2.06	61.36 ± 2.72
HC, mm	214.42 ± 5.28	221.07 ± 6.06	226.80 ± 7.59
AC, mm	190.81 ± 7.75	196.47 ± 7.94	202.38 ± 7.77
FL, mm	41.19 ± 1.63	42.30 ± 1.75	43.26 ± 1.98
BPD for Z-score	−1.14 ± 0.82	0.10 ± 0.74	1.27 ± 0.83
HC for Z-score	−1.02 ± 0.81	0.21 ± 0.75	1.54 ± 0.77
AC for Z-score	−0.68 ± 0.92	0.22 ± 0.91	1.34 ± 0.79
FL for Z-score	−0.99 ± 1.04	−0.15 ± 0.89	0.77 ± 1.10
Ultrasound parameters at 30 weeks of gestation			
BPD, mm	75.59 ± 2.77	77.81 ± 3.06	80.58 ± 2.85
HC, mm	279.52 ± 9.72	285.06 ± 9.85	295.02 ± 9.87
AC, mm	260.15 ± 11.59	265.87 ± 13.15	277.22 ± 12.22
FL, mm	56.61 ± 2.68	57.77 ± 2.71	59.41 ± 2.18
BPD for Z-score	−0.77 ± 0.80	0.15 ± 0.82	1.11 ± 0.79
HC for Z-score	−0.50 ± 0.78	0.23 ± 0.77	1.27 ± 0.83
AC for Z-score	−0.30 ± 0.79	0.33 ± 0.84	1.29 ± 0.98
FL for Z-score	−0.31 ± 1.06	0.43 ± 1.04	1.17 ± 1.08
Ultrasound parameters at 37 weeks of gestation			
BPD, mm	89.41 ± 2.52	91.53 ± 2.54	93.30 ± 2.33
HC, mm	324.14 ± 8.56	329.74 ± 8.42	335.40 ± 8.53
AC, mm	324.37 ± 13.12	330.52 ± 11.98	336.37 ± 12.06
FL, mm	69.36 ± 2.24	69.93 ± 1.94	70.99 ± 2.11
BPD for Z-score	−0.55 ± 0.82	0.18 ± 0.79	0.78 ± 0.82
HC for Z-score	−0.52 ± 0.88	0.10 ± 0.85	0.73 ± 0.84
AC for Z-score	−0.55 ± 0.78	−0.12 ± 0.75	0.28 ± 0.71
FL for Z-score	−0.64 ± 0.84	−0.38 ± 0.75	0.07 ± 0.89
Birth weight, g			
	3200.07 ± 353.41	3339.24 ± 386.80	3552.04 ± 345.17

All comparisons were made between groups by Analysis of Variance (ANOVA) and all the *p* values were <0.001. Pairwise comparisons of birth weight were performed using *post-hoc* tests with Bonferroni correction: consistently low v.s moderate: *p* = 0.001, consistently low vs. high-falling: *p* < 0.001, moderate vs. high-falling: *p* < 0.001. BPD, biparietal diameter; HC, head circumference; AC, abdominal circumference; FL, femur length.

The associations between single birth weight measurements and neonatal mtDNAcn are presented in Table 3. No statistically significant associations of birth weight with the percentage change in newborn mtDNAcn were observed.

**Table 3:** Association of birth weight with the percentage change in newborn mtDNAcn in cord blood.

Participants	Unadjusted Model		Adjusted Model	
	percent change (95% CI)^a^	*p* value	percent change (95% CI)^a^	*p* value
All participants^b^	−0.46 (−17.01, 19.40)	0.961	0.46 (−17.59, 22.74)	0.956
Male newborns^c^	4.23 (−17.21, 31.22)	0.821	9.40 (−14.89, 40.60)	0.484
Female newborns^c^	−7.10 (−30.18, 23.88)	0.620	−10.67 (−34.54, 21.90)	0.477

^a^ Data are presented as the percentage change (95% CI) in newborn mtDNAcn per 1,000 g increase in birth weight.

^b^ Model was adjusted for maternal age, pre-pregnancy BMI, maternal education level, parity, GDM, PIH, passive smoking during pregnancy, newborn sex, and gestational age at birth.

^c^ Model was adjusted for maternal age, pre-pregnancy BMI, maternal education level, parity, GDM, PIH, passive smoking during pregnancy, and gestational age at birth.

mtDNAcn, mitochondrial DNA copy number; BMI, body mass index; PIH, pregnancy-induced hypertension; GDM, gestational diabetes mellitus.

Table 4 shows the relationship between individual fetch ultrasound parameters and mtDNAcn. Only a positive correlation was found between FL Z-score and mtDNAcn at 37 weeks of pregnancy, meaning that for every unit increase in FL Z-score, mtDNAcn increased by 9.60% (95% CI: 0.55%, 19.47%; *p* = 0.037). In the results of sex stratification, only statistically significant results were found in boys, with AC Z-score (*β* = 15.59%; 95% CI: 2.11%, 30.85%; *p* = 0.022) and FL Z-score (*β* = 17.00%; 95% CI: 4.08%, 31.52%; *p* = 0.009) at 37 weeks of pregnancy positively correlated with mtDNAcn.

**Table 4:** Association of fetal ultrasound measurement Z-scores with the percentage change in newborn mtDNAcn in cord blood.

Ultrasound measurements	All participants^b^		Male newborns^c^		Female newborns^c^	
	Percent change (95% CI)^a^	*p* value	Percent change (95% CI)^a^	*p* value	Percent change (95% CI)^a^	*p* value
16 weeks of gestation						
BPD for Z-score	3.91 (−3.37, 11.73)	0.300	3.31 (−5.88, 13.40)	0.491	3.85 (−7.43, 16.50)	0.518
HC for Z-score	5.70 (−0.91, 12.75)	0.092	5.95 (−2.61, 15.26)	0.178	3.36 (−6.62, 14.42)	0.521
AC for Z-score	1.27 (−5.52, 8.55)	0.721	−1.14 (−9.68, 8.22)	0.803	1.38 (−9.04, 13.00)	0.803
FL for Z-score	0.08 (−6.19, 6.76)	0.981	3.25 (−5.45, 12.75)	0.475	−1.54 (−10.63, 8.48)	0.753
24 weeks of gestation						
BPD for Z-score	−1.50 (−8.02, 5.47)	0.664	−1.63 (−10.36, 7.95)	0.728	−1.09 (−10.88, 9.78)	0.837
HC for Z-score	−3.08 (−9.40, 3.68)	0.363	−4.41 (−12.82, 4.80)	0.335	−1.82 (−11.40, 8.79)	0.724
AC for Z-score	−0.60 (−7.33, 6.62)	0.866	−2.46 (−11.75, 7.80)	0.624	−0.63 (−10.22, 9.99)	0.902
FL for Z-score	−0.26 (−6.65, 6.58)	0.939	2.53 (−6.00, 11.85)	0.571	−3.07 (−12.63, 7.54)	0.555
30 weeks of gestation						
BPD for Z-score	2.33 (−5.40, 10.68)	0.565	3.35 (−7.06, 14.91)	0.542	1.82 (−9.81, 14.94)	0.770
HC for Z-score	3.03 (−5.10, 11.85)	0.476	6.32 (−4.10, 17.87)	0.243	−0.99 (−13.90, 13.86)	0.889
AC for Z-score	−3.31 (−10.98, 5.02)	0.424	−3.86 (−13.09, 6.35)	0.443	−2.88 (−15.80, 12.03)	0.687
FL for Z-score	3.8 (−2.98, 11.06)	0.279	4.60 (−3.46, 13.34)	0.270	1.16 (−10.40, 14.23)	0.851
37 weeks of gestation						
BPD for Z-score	2.02 (−5.83, 10.52)	0.624	4.41 (−6.44, 16.53)	0.440	−1.64 (−12.70, 10.81)	0.784
HC for Z-score	3.53 (−4.21, 11.91)	0.381	10.16 (−0.82, 22.37)	0.071	−3.20 (−13.84, 8.77)	0.583
AC for Z-score	7.88 (−1.85, 18.58)	0.115	15.59 (2.11, 30.85)	0.022	−1.67 (−15.12, 13.91)	0.822
FL for Z-score	9.60 (0.55, 19.47)	0.037	17.00 (4.08, 31.52)	0.009	3.42 (−9.22, 17.83)	0.611

^a^ Data are presented as the percentage change (95% CI) in newborn mtDNAcn.

^b^ Model was adjusted for maternal age, pre-pregnancy BMI, maternal education level, parity, GDM, PIH, passive smoking during pregnancy, newborn sex, and gestational age at birth.

^c^ Model was adjusted for maternal age, pre-pregnancy BMI, maternal education level, parity, GDM, PIH, passive smoking during pregnancy, and gestational age at birth.

mtDNAcn, mitochondrial DNA copy number; BMI, body mass index; PIH, pregnancy-induced hypertension; GDM, gestational diabetes mellitus; Biparietal diameter, BPD; head circumference, HC; abdominal circumference, AC; femur length, FL.

Table 5 shows the percentage change in mtDNAcn for the alternative fetal growth patterns compared with the “moderate” pattern. The association between the “consistently low” pattern and decreased mtDNAcn was observed exclusively in male newborns, with a 24.32% (95% CI: −40.57%, −3.62%; *p* = 0.026) reduction in mtDNAcn in the unadjusted model. This association remained stable in the adjusted models (*β* = −22.91%; 95% CI: −39.33%, −1.83%; *p* = 0.039). Table 6 shows the association of fetal growth trajectories and newborn mtDNAcn tertiles when mtDNAcn was divided into three groups (the first, second, and third tertile). When using the third tertile as a reference, boys exhibiting a “consistently low” pattern were more likely to fall within the first tertile of mtDNAcn compared to those with a “moderate” pattern (OR = 2.44; 95% CI: 1.09, 5.45; *p* = 0.030).

**Table 5:** Association of fetal growth trajectory with the percentage change in newborn mtDNAcn in cord blood.

Fetal growth trajectory	Unadjusted Model		Adjusted Model	
	Percent change (95% CI)^a^	*p* value	Percent change (95% CI)^a^	*p* value
All participants^b^				
Consistently low	−13.50 (−26.89, 2.09)	0.087	−11.90 (−25.70, 4.23)	0.139
Moderate	0.00 (Reference)		0.00 (Reference)	
High-falling	−8.59 (−23.97, 10.15)	0.345	−8.17 (−23.79, 10.66)	0.366
Male newborns^c^				
Consistently low	−23.97 (−40.43, −3.17)	0.026	−22.55 (−39.19, −1.37)	0.039
Moderate	0.00 (Reference)		0.00 (Reference)	
High-falling	−15.86 (−32.55, 4.71)	0.112	−14.89 (−31.77, 6.41)	0.157
Female newborns^c^				
Consistently low	−3.17 (−23.62, 22.74)	0.789	−2.73 (−23.09, 23.03)	0.817
Moderate	0.00 (Reference)		0.00 (Reference)	
High-falling	3.28 (−25.53, 42.89)	0.850	0.69 (−27.05, 39.32)	0.961

^a^ Data are presented as the percentage change (95% CI) in newborn mtDNAcn compared with the “moderate” group.

^b^ Model was adjusted for maternal age, pre-pregnancy BMI, maternal education level, parity, GDM, PIH, passive smoking during pregnancy, newborn sex, and gestational age at birth.

^c^ Model was adjusted for maternal age, pre-pregnancy BMI, maternal education level, parity, GDM, PIH, passive smoking during pregnancy, and gestational age at birth.

mtDNAcn, mitochondrial DNA copy number; BMI, body mass index; PIH, pregnancy-induced hypertension; GDM, gestational diabetes mellitus.

**Table 6:** Association of fetal growth trajectory with newborn mtDNAcn tertiles in cord blood.

Fetal growth trajectory	First tertile (∼0.8726)	*p*-value	Second tertile (0.8727∼1.7636)	*p*-value
All participants^a^				
Consistently low	1.79 (1.09, 2.96)	0.022	1.08 (0.64, 1.82)	0.782
Moderate	1.00 (Reference)		1.00 (Reference)	
High-falling	1.19 (0.68, 2.07)	0.541	0.92 (0.53, 1.59)	0.761
Male newborns^b^				
Consistently low	2.44 (1.09, 5.45)	0.030	1.41 (0.64, 3.12)	0.395
Moderate	1.00 (Reference)		1.00 (Reference)	
High-falling	1.88 (0.91, 3.86)	0.086	0.98 (0.49, 1.96)	0.947
Female newborns^b^				
Consistently low	1.40 (0.72, 2.70)	0.320	0.90 (0.43, 1.88)	0.773
Moderate	1.00 (Reference)		1.00 (Reference)	
High-falling	0.59 (0.22, 1.55)	0.280	0.91 (0.35, 2.36)	0.844

^a^ Model was adjusted for maternal age, pre-pregnancy BMI, maternal education level, parity, GDM, PIH, passive smoking during pregnancy, newborn sex, and gestational age at birth.

^b^ Model was adjusted for maternal age, pre-pregnancy BMI, maternal education level, parity, GDM, PIH, passive smoking during pregnancy, and gestational age at birth.

The third tertile (1.7637∼) was designated as the reference group in all regression models. mtDNAcn, mitochondrial DNA copy number; BMI, body mass index; PIH, pregnancy-induced hypertension; GDM, gestational diabetes mellitus.

## 4 Discussion

In this study, we utilized serial ultrasound measurements throughout pregnancy to identify three distinct patterns of intrauterine fetal growth trajectory combined BPD, HC, FL, and AC. We found that the “consistently low” pattern was associated with reduced cord blood mtDNAcn in male newborns. Even after adjusting for potential confounding factors, this association remained significant.

Previous studies have suggested that neonates may exhibit distinct patterns of intrauterine growth despite having similar birth sizes (2, 3). Unlike birth weight, which indirectly reflects intrauterine growth, ultrasound measurements during pregnancy provide a direct reflection of fetal growth *in utero*. Serial fetal ultrasonography, as a routine procedure during prenatal care, offers a convenient and effective means of capturing fetal development at different gestational stages. As demonstrated by many epidemiological studies, the fetal growth trajectory derived from serial ultrasonic measurements presents a dynamic pattern of intrauterine growth, which has been recognized for its association with health outcomes not only in childhood (28, 30–33) but also throughout adulthood (34–38). For example, fetuses exposed to adverse intrauterine environments may adopt a subnormal growth trajectory to increase their chances of survival. However, such developmental reprogramming can increase the risk of metabolic diseases in adulthood (39, 40). The Raine Study conducted in Western Australia suggested that restricted fetal AC and HC trajectory patterns described as “low-falling” or “low-stable” patterns were associated with elevated systolic blood pressure (38) and insulin resistance (36) over a span of two decades. However, no studies have proposed early-life biomarkers associated with below-normal intrauterine growth patterns.

In this study, we conducted an association analysis between different fetal growth trajectories and mtDNAcn. Our findings indicated that, compared with fetuses exhibiting a “moderate growth” trajectory, male infants with a “consistently low” trajectory were more likely to have reduced mtDNAcn in cord blood. In the context of pregnancy, the mtDNA content in placental and umbilical cord blood has been studied as a potential biomarker for growth and development (13–19).

Several studies have explored the association between birth weight and placental or cord blood mtDNA content. For example, Gemma C et al. (14) used the “mtDNA/nuclear DNA ratio” as a research indicator and reported that the mtDNA content in cord blood was lower in small-for-gestational-age (SGA, birthweight-for-gestational-age is less than the 10th percentile) infants than in appropriate-for-gestational-age (AGA, birthweight-for-gestational-age is between the 10th and 90th percentiles) infants (18 ± 6 vs. 28 ± 4, *p* < 0.03). Findings from the INMA birth cohort (17, 18) suggested that exposure to air pollution during pregnancy was associated with lower birth weight (a decrease of 46.7 g in birth weight for each 10 µg/m^3^ increase in NO_2_), and placental mitochondrial content served as a mediator variable with a mediating proportion of 10% and an indirect effect of −5.2 g. Guitar-Mampel M. et al. (15) studied IUGR newborns and reported a 5.62% decrease in the mtDNA content in their cord blood compared with that of normal birth weight newborns. However, the difference was not statistically significant, and the study included a small sample size of 14 IUGR infants and 22 normal birth weight infants. In contrast, two studies reported increased placental mtDNA content in IUGR newborns (13, 16). The inconsistent results among these studies might be due to the pathological state of IUGR, where the placenta undergoes “reprogramming” in terms of energy metabolism to maximize intrauterine growth satisfaction for the fetus (41). In this study, we did not observe a statistically significant positive or negative association between cord blood mtDNAcn and birth weight. This could be attributed to the absence of IUGR cases with pathological conditions in our study population, as all the newborns had normal birth weights.

Considering that different fetal growth patterns can exist even among infants with the same birth weight (2, 3), we examined the impact of these patterns on neonatal mtDNAcn in a healthy population with birth weights ≥ 2,500 g. To the best of our knowledge, this is the first study to report a correlation between fetal growth patterns of “consistently low” and mtDNA depletion, which is considered a putative link between early-life health and adverse metabolic outcomes in adulthood (14). The potential underlying mechanism for this correlation is that alterations in mitochondrial function could serve as early indicators of impaired fetal growth resulting from adverse intrauterine conditions (42), even if these alterations do not result in low birth weight. Hence, on the basis of the accumulated evidence of fetal growth patterns and postnatal metabolic health (35–38), we hypothesize that mtDNA depletion may be a potential link between adverse fetal growth patterns and increased risk factors for metabolic disease later in life. It is noteworthy that statistically significant results were exclusively observed in males. This is attributed to the fact that male fetuses exhibit greater sensitivity to oxidative stress (43) and responses to DNA damage (44).

There are several strengths in our study. First, the findings of this research were progressive and novel in light of the existing body of literature, as this was the first study to explore the associations of fetal growth patterns with neonatal mtDNAcn. Our study examined fetal growth throughout pregnancy, not just birth weight. Second, we utilized standardized, continuous ultrasound measurements during pregnancy, which are considered integral components in endeavors aimed at preventing metabolic health-related diseases during early life stages (45, 46). Fetal ultrasound measurement during pregnancy is a convenient and accessible technique, even in underdeveloped areas (6). Thus, prenatal care interventions based on our findings can be applied to a greater extent. Third, we performed strict quality control for mtDNAcn detection, which made the results more credible.

However, this study also has several limitations. First, the participants were all from a single ethnic group, which limits the generalization of the research results to other ethnic groups. Second, although our model adjusted for several covariates, residual or unmeasured confounding factors (e.g., maternal dietary) may still influence our results. Finally, future research is needed to elucidate the mechanisms driving unique fetal growth patterns during pregnancy and understand how adverse patterns increase the risk of metabolic diseases.

## 5 Conclusions

In summary, our research identified three distinct fetal growth trajectories in normal birth weight infants and revealed that the “consistently low” trajectory was associated with reduced neonatal mtDNAcn in male infants. On the basis of accumulating evidence, we propose that mtDNA depletion serves as one of the presumptive links between adverse fetal growth patterns and an increased risk of metabolic diseases after birth. Our findings reaffirm the critical role of fetal growth and intrauterine development data in preventing metabolic health issues in early life.

## Data Availability

The datasets presented in this article are not readily available because Data are available on reasonable request. The data set is available from the corresponding author on reasonable request and if consistent with the projects's ethics approvals. Requests to access the datasets should be directed to Kai Chen, chenkai@zgwhfe.com.
